# Short-term clinical outcomes and five-year survival analysis of laparoscopic-assisted transanal natural orifice specimen extraction versus conventional laparoscopic surgery for sigmoid and rectal cancer: a single-center retrospective study

**DOI:** 10.3389/fsurg.2023.1340869

**Published:** 2024-01-03

**Authors:** Zhizhong Zheng, Fenfen Kang, Yugang Yang, Yicong Fang, Kaiyuan Yao, Qunzhang Zeng, Muhai Fu, Lixiong Luo, Xiajuan Xue, Shuijie Lin, Xingpeng Shi, Xun Fang, Baohua Zhou, Yincong Guo

**Affiliations:** ^1^ Department of Colorectal and Anal Surgery, Zhangzhou Affiliated Hospital of Fujian Medical University, Zhangzhou, China; ^2^ Department of Anesthesiology, Zhangzhou Affiliated Hospital of Fujian Medical University, Zhangzhou, China

**Keywords:** laparoscopy, survival analysis, intraabdominal infections, colorectal surgery, sigmoid

## Abstract

**Background:**

The cosmetic benefits of natural orifice specimen extraction (NOSE) are easily noticeable, but its principles of aseptic and tumor-free procedure have caused controversy.

**Methods:**

We conducted a retrospective analysis of the clinical data of patients who underwent laparoscopic-assisted transanal NOSE or conventional laparoscopic surgery (CLS) for sigmoid and rectal cancer at our hospital between January 2018 and December 2018. The study aimed to compare the general characteristics, perioperative indicators, postoperative complications, and five-year follow-up results between the two groups.

**Results:**

A total of 121 eligible patients were enrolled, with 52 underwent laparoscopic-assisted transanal NOSE and 69 underwent CLS. There were no significant differences observed between the two groups in terms of gender, age, body mass index (BMI), TNM stage, etc. (*P* > 0.05). However, the NOSE group exhibited significantly shorter total incision length and longer operation time compared to the CLS group (*P* < 0.05). There were no statistically significant differences observed between the two groups in terms of positive rate of bacterial culture, incidence rates of intraabdominal infections or anastomotic leakage (*P* > 0.05). Furthermore, during follow-up period there was no statistically significant difference observed between these two groups concerning overall survival rate and disease-free survival outcomes (*P* > 0.05).

**Conclusions:**

The management of surgical complications in CLS is exemplary, with NOSE presenting a sole advantage in terms of incision length albeit at the cost of prolonged operative time. Therefore, NOSE may be deemed appropriate for patients who place high emphasis on postoperative cosmetic outcomes.

## 1. Introduction

Despite the gradual acceptance of early screening for colorectal tumors, the global incidence and mortality rates of colorectal cancer remain alarmingly high, currently ranking third in terms of incidence and second in terms of mortality worldwide (1), this persistent trend poses significant challenges to colorectal surgeons. Surgical intervention represents the foremost and efficacious modality for managing colorectal neoplasms (2, 3). The transition from open surgery to laparoscopic surgery represents a groundbreaking milestone in the management of colorectal cancer. Modern surgeons strive not only for radical tumor cure but also for minimizing surgical trauma. Moreover, extensive evidence supports the safety and efficacy of laparoscopic colorectal surgery, which is associated with smaller incisions, faster postoperative recovery, and even improved tumor prognosis compared to open surgery (4–6). Consequently, it has gained widespread utilization in clinical practice. However, laparoscopic surgery inevitably necessitates a lengthy abdominal incision for specimen extraction and digestive tract reconstruction. This lengthy incision has led to numerous complications associated with wounds, including infection and hernia formation, which contradicts the fundamental principle of minimally invasive surgery. In the pursuit of achieving a more minimally invasive approach, the emergence of NOSE has revolutionized surgeons' perspectives. By utilizing the natural cavity passage for specimen retrieval, it eliminates the need for lengthy abdominal incisions, resulting in reduced trauma and enhanced aesthetic outcomes. Since its introduction by Franklin et al. (7) in 1993, who reported a series of patients undergoing laparoscopic sigmoid colon resection with transanal specimen removal, this technique has gained widespread recognition and adoption in China (8).

Despite the numerous advantages associated with NOSE, its principle of aseptic and tumor-free procedure remains a subject of controversy (9). The intraperitoneal opening of the intestinal cavity poses an increased risk for intraperitoneal infection and tumor dissemination, whereas extraction of specimens through the natural duct may potentially lead to rectal stump implantation and metastasis. Some studies pertaining to the NOSE have indeed substantiated its safety; however, there exists a dearth of outcomes derived from bacterial culture analysis of postoperative abdominal drainage fluid. Furthermore, the majority of these investigations suffer from limited availability of data. Meanwhile, the limited duration of NOSE surgery and the lack of long-term survival analysis preclude a comprehensive assessment of the efficacy of NOSE cancer treatment (10). This retrospective study aimed to investigate the short-term clinical outcomes and five-year follow-up of laparoscopic-assisted transanal NOSE compared to CLS for the treatment of sigmoid and rectal cancer.

## 2. Materials and methods

**Figure 1:**
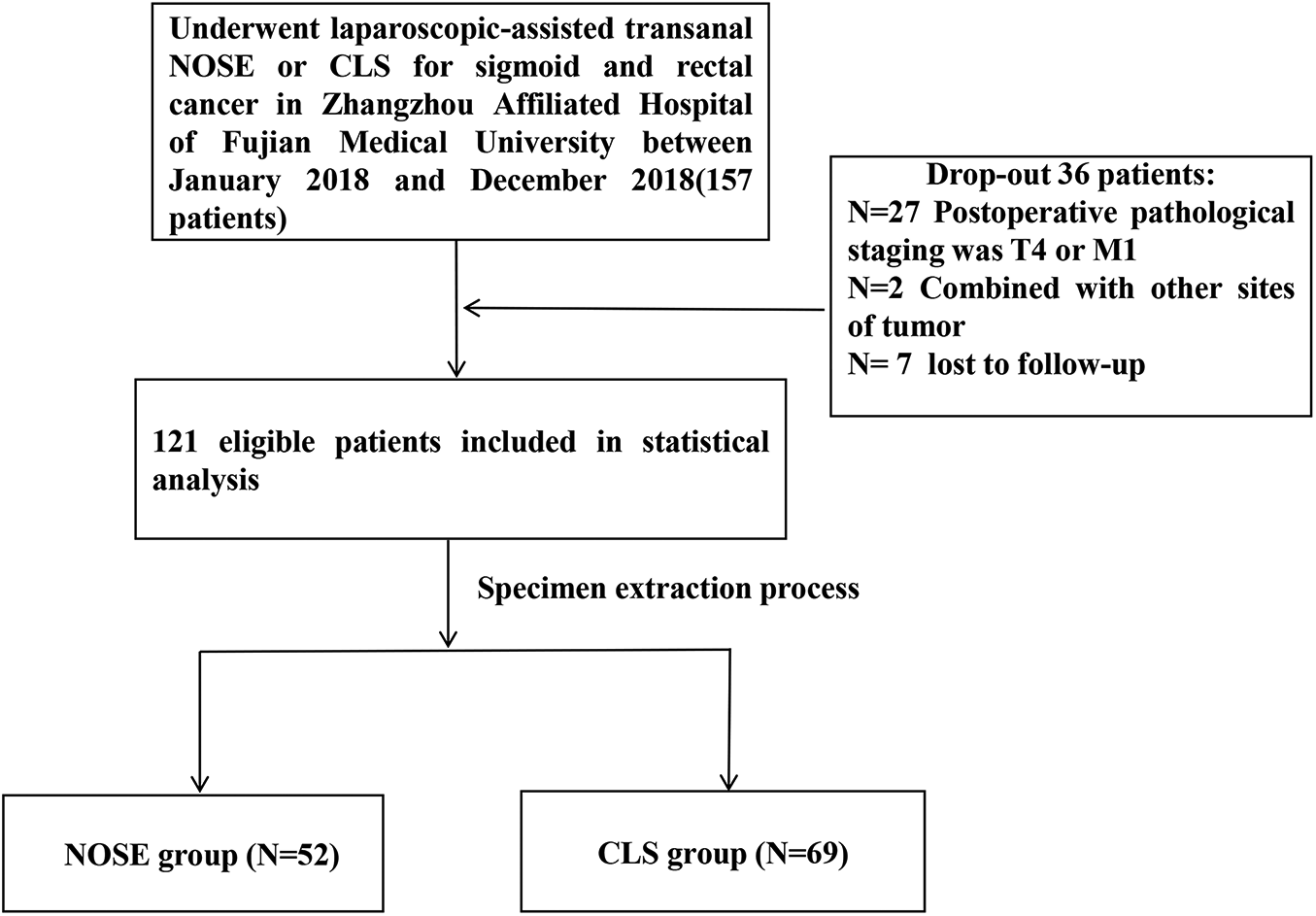
Patient recruitment process. NOSE, natural orifice specimen extraction; CLS, conventional laparoscopic surgery.

This retrospective study (Registration No. 2020LWB035) was conducted at Zhangzhou Affiliated Hospital of Fujian Medical University, with approval from the ethics committee and informed consent obtained from all patients involved. The inclusion criteria encompassed: (1) patients who underwent laparoscopic-assisted transanal NOSE or CLS for sigmoid and rectal cancer between January 2018 and December 2018 at our institution; (2) patients with confirmed diagnoses of sigmoid or rectal cancer through preoperative colonoscopy and pathology assessments; (3) patients classified as T_0–3_N_0–2_M_0_ stage based on CT or MRI evaluations prior to surgery; (4) patients without evidence of distant metastasis or invasion into adjacent organs; and finally; (5) patients without any concurrent malignant tumors or significant systemic diseases such as cardiac, hepatic, renal conditions, among others. The exclusion criteria were as follows: (1) patients who had to undergo open surgery due to the discovery of distant metastasis or invasion of adjacent organs during the operation; (2) patients who were unable to provide complete follow-up data after surgery. Based on the different surgical methods, the included patients were divided into two groups: NOSE group and CLS group. The general characteristics, perioperative indicators, postoperative complications, and five-year follow-up results of these two groups were compared (Figure 1).

### 2.1. Preoperative preparation and anesthesia

All patients were given a prescription for metronidazole tablets two days prior to their surgery and received oral laxatives the evening before the procedure to prepare their bowels. Before the surgery, a single infusion of cefmetazole was routinely administered intravenously 30 min beforehand. If the surgical procedure lasted longer than 3 h, the same dosage was repeated. General anesthesia was uniformly administered to all patients during anesthetic induction.

### 2.2. Surgical intervention

The patient underwent a modified lithotomy position and achieved pneumoperitoneum at 13 mmHg. The abdominal wall was punctured with five trocars: one 10-mm camera port above the navel, one 12-mm surgeon's operation port in the lower right quadrant, two 5-mm ports on the left side aligned with the spina iliaca anterior superior in both middle and lower abdomen, and one 5-mm port on the right side in the middle abdomen. Tumor and lymphoid tissue dissection were conducted following total mesorectal excision (TME). Sigmoidectomy was performed for tumors located in the sigmoid colon, anterior resection was performed for tumors located in the upper rectum, and low anterior resection was performed for tumors located in the lower rectum.

The CLS group underwent a conventional laparoscopic-assisted procedure for radical sigmoidectomy or proctectomy, involving the creation of a hypogastric incision measuring 4–6 cm in length. After separating the mesocolon, they proceeded to divide the proximal colon and remove tumor tissue. Subsequently, an anvil was introduced into the distal colon to facilitate bowel anastomosis.

The NOSE group received treatment using CRC-NOSES VI (11). A linear cutter stapler was utilized to divide the proximal 10 cm of the colon tumor and the lower edge of the tumor (up to 2–3 cm for rectal cancer and 10 cm for sigmoid colon cancer). Following that, a thorough disinfection of the rectal lumen was conducted using diluted povidone-iodine, followed by an incision in the rectum. Subsequently, a sterile protective sleeve was inserted into the abdominal cavity through which excised diseased tissue could be safely extracted along with the protective sleeve. Next, the circular stapling device's anvil was inserted into the abdominal cavity through the rectal stump. Sterile gauze was carefully placed around the proximal colon. A precise longitudinal incision of approximately 2 cm was made on the wall of the proximal colon to allow for insertion of the anvil in this area. Finally, using an endoscopic linear stapler, both the exposed proximal colon and rectal stump were expertly closed.

In both study groups, the circular stapling device was meticulously inserted into the rectum, followed by a laparoscopic-guided end-to-end anastomosis with the anvil junction positioned in the proximal colon. Subsequently, thorough irrigation of the abdomen and pelvis was performed using a substantial volume of normal saline solution, while concurrently placing a pelvic drainage tube. On postoperative day one, peritoneal drainage fluid samples were collected for bacterial culture analysis.

### 2.3. Follow-up

According to the guidelines provided by the NCCN, adjuvant chemotherapy was administered to all patients who had undergone surgery for T3/T4 or postoperative node-positive tumors. Follow-up appointments were scheduled every 3–6 months within the first three years, which included physical examinations and laboratory tests incorporating tumor biomarkers such as CEA and CA-199. Biannual CT scans of the chest, abdomen, and pelvis were performed, while a complete colonoscopy was planned on an annual basis. The patients were observed at intervals of 6–12 months after the surgery, either through outpatient visits or telephone communication, until the occurrence of CRC recurrence and metastasis or October 01, 2023. The main goals of this study were to assess the long-term outcomes of overall survival (OS) and disease-free survival (DFS) over a period of five years. This approach is in line with the stringent standards expected by Nature journal for scholarly writing.

### 2.4. Statistical analysis

The statistical data were processed using SPSS software version 27.0 for Windows (IBM Corp., Armonk, NY, United States). Quantitative variables were analyzed utilizing the Student's *t*-test and expressed as mean ± standard deviation (SD). Categorical variables were presented as a percentage (%) and compared employing Pearson's Chi-Square (χ^2^) test or Fisher's exact test when appropriate. The Kaplan–Meier method was employed to calculate survival outcomes of patients in both groups, and differences in survival curves (OS and DFS) were compared through the log-rank test. A significance level of *P *< 0.05 was considered statistically significant according to established conventions.

## 3. Results

### 3.1. The clinical characteristics of the participants

The CLS group comprised a total of 39 males and 30 females, with an average age of 60.7 ± 11.4 years. Similarly, the NOSE group consisted of 28 males and 24 females, with an average age of 62.2 ± 10.0 years. There were no statistically significant differences in clinical characteristics between the NOSE and CLS groups, including age, gender, BMI, history of abdominal operations, and metastasis (TNM) stages (*P* > 0.05; Table 1).

**Table 1:** Patient characteristics.

Clinical characteristics	NOSE group (*n* = 52)	CLS group (*n* = 69)	*t*/χ^2^	*P*
Age (years)	62.2 ± 10.0	60.7 ± 11.4	0.747	0.457
Gender			0.086	0.769
Male	28	39		
Female	24	30		
BMI (kg/m^2^)	22.7 ± 3.2	21.9 ± 3.4	1.468	0.145
Abdominal operation history			0.268	0.605
Presence	6	6		
Absence	46	63		
TNM stages			5.651	0.130
0	7	2		
Ⅰ	11	13		
Ⅱ	17	23		
Ⅲ	17	31		

Data are shown as mean ± SD or *n*.

### 3.2. Perioperative outcomes

No conversions to open surgery were observed, and there were no incidences of incision infection. When comparing NOSE with CLS group, significant differences were noted in the effect on operation time (213.9 ± 20.0 min vs. 194.1 ± 20.6 min, *t* = 5.292, *p* *<* 0.01) and total incision length (7.0 ± 0.0 cm vs. 11.7 ± 0.8 cm, *t* = −12.435, *p* *<* 0.01). However, the differences between the groups regarding positive rate of bacterial culture (15.4% vs. 8.7%, χ^2^ = 1.297, *p *= 0.255) and intraabdominal infections (9.6% vs. 2.9%, χ^2^ = 2.455, *P *= 0.117) did not reach statistical significance. Eight patients in the NOSE group tested positive for bacterial culture; among them, five patients had escherichia coli cultured from drainage fluid. Six patients in the CLS group tested positive for bacterial culture and five patients had escherichia coli cultured from drainage fluid as shown in Table 2.

**Table 2:** Operative and postoperative outcomes.

Perioperative outcomes	NOSE group (*n* = 52)	CLS group (*n* = 69)	*t*/χ^2^	*P*
Operation time (min)	213.9 ± 20.0	194.1 ± 20.6	5.292	<0.01
Intraoperative blood loss (ml)	32.1 ± 13.3	38.6 ± 15.4	−2.412	0.051
Total incision length (cm)	7.0 ± 0.0	11.7 ± 0.8	−12.435	<0.01
No. of lymph nodes retrieved	18.0 ± 9.5	23.3 ± 8.9	−3.139	0.345
Duration for analgesic (days)	3.3 ± 0.5	3.42 ± 0.6	−0.592	0.558
Duration for the first postoperative exhaust (days)	2.8 ± 2.1	2.9 ± 2.3	−0.292	0.771
Duration for the first postoperative defecation (days)	4.1 ± 2.9	4.0 ± 2.4	0.130	0.991
Length of postoperative stay in hospital (days)	8.6 ± 6.7	7.8 ± 2.7	0.883	0.256
Postoperative complications (%)	17.4	10.1	1.326	0.250
Positive rate of bacterial culture (%)	15.4	8.7	1.297	0.255
Intraabdominal infection (%)	9.6	2.9	2.455	0.117
Anastomotic leakage (%)	3.8	1.4	0.705	0.401
Reoperation (%)	9.6	8.7	0.030	0.862

Data are shown as mean ± SD or %.

### 3.3. Survival analysis

**Figure 2:**
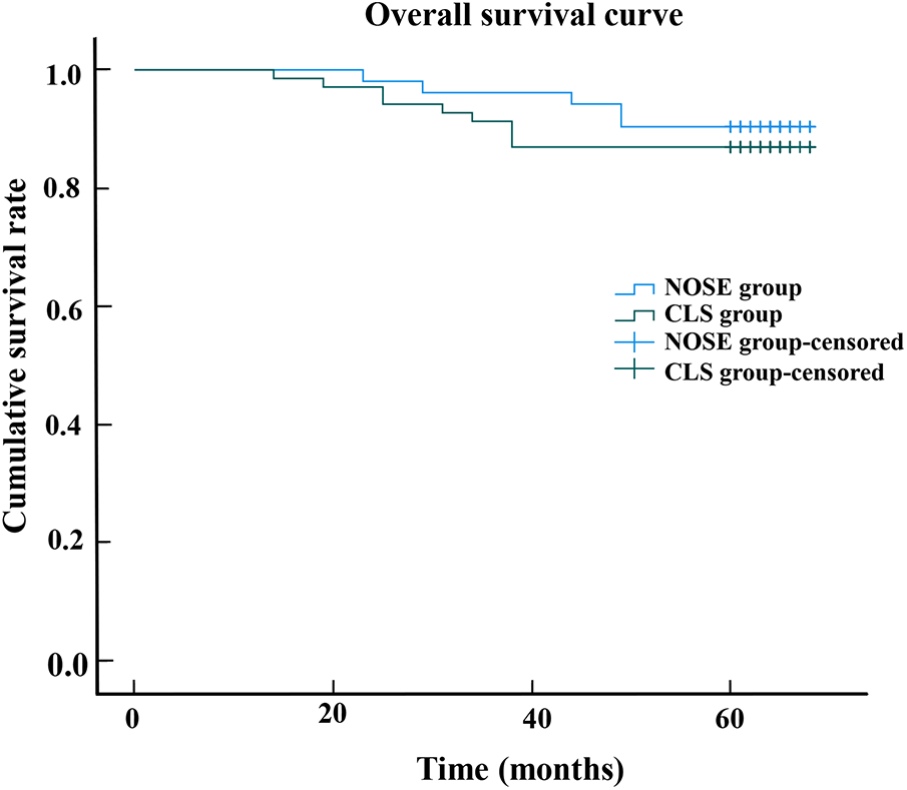
The overall survival curve shows that 5-year overall survival rate in the NOSE group and CLS group were 90.4% and 87.0%, respectively. There was no significant difference between the NOSE and CLS groups (*p* = 09.531).

**Figure 3:**
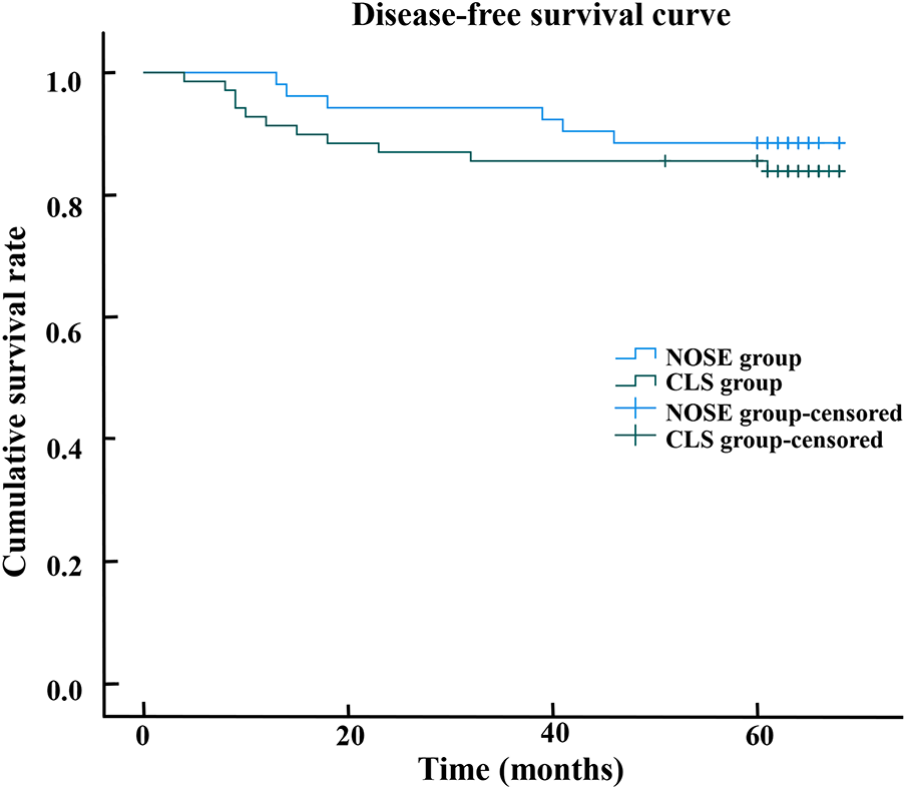
The disease-free survival curve shows that 5-year disease-free survival rate in the NOSE group and CLS group were 88.5% and 84.1%, respectively. There was no significant difference between the NOSE and CLS groups (*p* = 0.460).

The median follow-up period was 64.0 months (range, 14–68). Throughout the entire follow-up duration, a total of 14 out of the initial cohort of 121 patients succumbed to mortality, while an additional 17 patients experienced either local recurrence or distant metastasis. Notably, there was no statistically significant disparity observed in terms of tumor recurrence between the NOSE group and the CLS group. Within the NOSE group specifically, one patient exhibited local recurrence and five patients encountered distant recurrence following a median follow-up period of 64 months (range, 23–68). Conversely, within the CLS group, one patient demonstrated local recurrence and ten patients manifested distant recurrence after being monitored for a median follow-up duration of 63 months (range, 14–68). Only one patient in the NOSE group experienced recurrence at the anastomotic site. The Kaplan curves revealed that the overall survival (*p* = 0.531) and disease-free survival (*p* = 0.460) of the NOSE group were comparable to those of the CLS group. In the NOSS group, the 5-year overall survival rate was 90.4% and disease-free survival rate was 88.5%, while in the CLS group, these rates were slightly lower at 87.0% and 84.1%, respectively (Figures 2, 3).

## 4. Discussion

In recent years, technological advancements and innovations in surgical instruments have facilitated the performance of surgeries with reduced incisions (12, 13). However, conventional laparoscopic colorectal cancer surgery inevitably entails a longer auxiliary incision for specimen extraction and reconstruction of the digestive tract. Compared to CLS, the key distinguishing feature of NOSE in colorectal surgery lies in its ability to extract specimens through natural orifices, perform complete intraperitoneal anastomosis, and avoid lengthy abdominal incisions (14–16). Patients undergoing NOSE experience enhanced pain management and reduced incidence of incision infections, among other notable benefits.

Numerous studies had conducted comparisons between NOSE and CLS, yielding invaluable insights for the clinical application in colorectal oncology. Studies had demonstrated that NOSE are predominantly conducted using laparoscopic techniques, which offer enhanced precision and obviate the need for lengthy surgical incisions. Consequently, this approach minimized surgical bleeding and did not prolong the duration of the operation (17). Our study demonstrated that the NOSE group exhibited significantly longer surgery times. Clearly, the laparoscopic procedure for NOSE entailed greater complexity, and certain patients undergoing this technique may encounter challenges in extracting specimens due to the narrow rectal cavity and pelvis, consequently leading to prolonged operative duration. Therefore, we propose conducting preoperative assessments for rhinoplasty patients to not only assess tumor dimensions but also evaluate pelvic measurements. As demonstrated by several studies, patients undergoing NOSE exhibit reduced reliance on postoperative analgesia and report lower pain scores (18–20). Additionally, NOSE has been shown to positively impact postoperative intestinal function recovery and lead to a decreased length of hospital stay (19, 21, 22). There may be multiple factors contributing to this outcome: (Ⅰ) The implementation of complete laparoscopic dissection and reconstruction of the digestive tract effectively minimizes excessive traction on the intestinal tract; (Ⅱ) The utilization of smaller incisions resulting in reduced postoperative pain enables patients to regain mobility earlier after surgery. Our study demonstrates comparable postoperative recovery outcomes among patients undergoing NOSE with CLS.

The postoperative abdominal or pelvic infection resulting from the dissemination of intestinal bacteria during bowel opening and anvil passage through the anorectum had garnered significant attention. Previous studies had substantiated this potential bacterial contamination following NOSE by assessing the prevalence of positive bacterial culture in intraoperative pelvic fluid (23, 24). Our research findings indicate that the predominant bacterial cultures in abdominal drainage were primarily escherichia coli, as a consequence of the dissemination of bacteria due to intestinal cavity opening. Numerous preventive measures had been implemented to impede the ingress of bacteria into the abdominal cavity, including ensuring meticulous bowel preparation, employing a linear cutter stapler for closure of both the proximal and lower edge of the tumor, irrigating with diluted 1% povidone-iodine prior to opening the rectal stump, and utilizing a sterile protective sleeve. Nevertheless, there remained an increased likelihood for bacterial dissemination through the aperture of the proximal bowel and rectal stump (25). However, it should be noted that not all instances of bacterial spread result in intraabdominal infections, and there were cases where patients with intraabdominal infections did not yield positive results in bacterial culture. Furthermore, our study revealed no significant disparity in celiac infections between the NOSE and CLS groups. Consequently, it is plausible to suggest that intracorporeal bowel opening did not augment the likelihood of abdominal or pelvic contamination. Additionally, patients did not encounter an extension in their hospital stay duration subsequent to receiving appropriate anti-infection treatment.

Another concern of the NOSE pertains to whether intraperitoneal dissection of the tumor bowel, opening of the rectal stump and proximal colon, and transrectal removal of the specimen result in exfoliation of cancer cells, potentially leading to recurrence in the abdominal and rectal stump. However, conclusive evidence regarding this matter is still lacking as only a limited number of studies have conducted comprehensive five-year survival analyses. The fundamental principle of tumor surgery is to achieve maximal resection, and the adoption of NOSE does not pose additional challenges in achieving complete tumor removal, particularly during lymph node dissection and mesangial separation. Notably, studies have demonstrated that both NOSE and CLS approaches yield comparable oncological outcomes over follow-up periods (20, 26, 27). Our study findings indicated that patients in the NOSE group demonstrated improved disease-free survival and overall survival outcomes compared to those in the CLS group; however, these differences did not reach statistical significance. We propose that this result may be attributed to limitations associated with CLS. Specifically, the vertical pull-out technique employed for excising diseased tissue through an abdominal incision, along with the compression of the incision protector, potentially increased the risk of tumor cells falling outside the protected area due to gravitational forces. Moreover, it is noteworthy that immediate removal of the incision protector following extraction of diseased tissue from the abdominal cavity was not performed. It was imperative to employ the incision protector for safeguarding the wound during the resection of the proximal colon and placement of the anvil of circular stapling device. However, this inadvertently facilitated tumor cell infiltration into the abdominal cavity, potentially leading to metastasis. In contrast, within the NOSE group, we utilized sterile protective sleeves to facilitate smooth specimen extraction and prevent tumor deposition at the open rectal stump. Subsequently, these sleeves were meticulously removed along with the specimen.

The study has certain limitations, as it did not employ a prospective design. Moreover, due to the restricted sample size, obtaining valid data on specific key findings was unattainable. For instance, no statistically significant difference was detected in bacterial culture results. Peritoneal drainage was only cultured for bacteria on the first day after surgery and was not continuously sampled to prevent potential false-negative results. Regrettably, further investigation into the correlation between bacterial culture in abdominal drainage fluid and intraperitoneal metastasis could not be conducted.

## 5. Conclusions

The management of surgical complications in CLS is exemplary, with NOSE presenting a sole advantage in terms of incision length albeit at the cost of prolonged operative time. Therefore, NOSE may be deemed appropriate for patients who place high emphasis on postoperative cosmetic outcomes.

## Data Availability

The raw data supporting the conclusions of this article will be made available by the authors, without undue reservation.
